# Adaptive Care and Remote Training: Models of Resilience for GWEPs During a Pandemic

**DOI:** 10.1093/geroni/igab046.1830

**Published:** 2021-12-17

**Authors:** Max Zubatsky

## Abstract

Service and training are interconnected for GWEP’s, whose dual missions are to advance training as well as service delivery to improve the care of older adults. The COVID-19 pandemic has necessitated a pivoting from in-person to remote delivery of program content and services. As a result, older adults and their families require the continuity of services with their providers due to the barriers that the pandemic has created. Additionally, universities and organizations have adapted virtually to teach learners how to work with older adults around different health initiatives. The pandemic required these programs to develop immediate services that provided an alternative to remote delivery services. This collection of GWEP programs utilized students and trainees in their older adult services and initiatives. The goal of this symposium is to demonstrate new models of educational and program delivery to enhance and extend training and service to new audiences. The symposium centers on best practices including technological tools to promote GWEP aims and will allow a discussion of challenges and outcomes faced. The session will be comprised of presentations from five university-based, Geriatric Workforce Enhancement Programs (approximately 12 minutes each), a 20 minute discussion and sharing of best practices, and a 10 minute question/answer session. Individual presentations will address areas that include: 1.) group interventions for dementia and caregivers, 2.) teaching interdisciplinary interns in conducting telehealth visits, 3.) adapting geriatrics regional conferences to reach older adults virtually, and 4.) Developing new programs and services for underserved and underrepresented older adult populations.

